# Down-regulation of COL1A1 inhibits tumor-associated fibroblast activation and mediates matrix remodeling in the tumor microenvironment of breast cancer

**DOI:** 10.1515/biol-2022-0776

**Published:** 2023-11-30

**Authors:** Bin Ma, Fangfang Li, Binlin Ma

**Affiliations:** Department of Breast and Thyroid Surgery, The Affiliated Cancer Hospital of Xinjiang Medical University, Urumqi 830011, Xinjiang, China; Department of Thyriod and Breast Surgery, West China School of Public Health, West China Fourth Hospital, Sichuan University, Chengdu 610041, China; Department of Breast and Thyroid Surgery, The Affiliated Cancer Hospital of Xinjiang Medical University, No. 789 Suzhou East Street, Urumqi 830011, Xinjiang, China

**Keywords:** fibroblasts, tumor microenvironment, matrix remodeling, collagen type I A1, CAV-1

## Abstract

We investigated the effects of collagen type I alpha 1 (COL1A1) on tumor-associated fibroblast activation and matrix remodeling in the tumor microenvironment of breast cancer. Cells were divided into the blank control, negative control, and siRNA-COL1A1 groups, or HKF control, HKF + exosomes (EXO), HKF + siRNA negative control-EXO, and HKF + siRNA-COL1A1-EXO co-culture groups. Western blot and quantitative real-time PCR detected gene expressions. COL Ⅰ, COL Ⅲ, and TGF-β1 were detected by enzyme-linked immunosorbent assay. We found that compared with blank and negative control groups, COL1A1 expression and the secretion of exosomes by breast cancer cells were inhibited in the siRNA-COL1A1 group. Compared with the HKF control group, the COL Ⅰ, COL Ⅲ, TGF-β1, α-SMA, and fibroblast activation protein (FAP) were increased, while the E-cadherin and CAV-1 were decreased in the HKF + EXO, HKF + siRNA negative control-EXO, and HKF + siRNA-COL1A1-EXO co-culture groups. Compared with HKF + EXO and HKF + siRNA negative control-EXO co-culture groups, the COL Ⅰ, COL Ⅲ, TGF-β1, α-SMA, and FAP were decreased, and the E-cadherin and CAV-1 were increased in the HKF + siRNA-COL1A1-EXO co-culture group. Collectively, COL1A1 down-regulation may inhibit exosome secretion possibly via inhibiting COL Ⅰ and upregulating CAV-1, thereby inhibiting tumor-associated fibroblast activation and matrix remodeling in the tumor microenvironment.

## Graphical abstract

**Figure:**
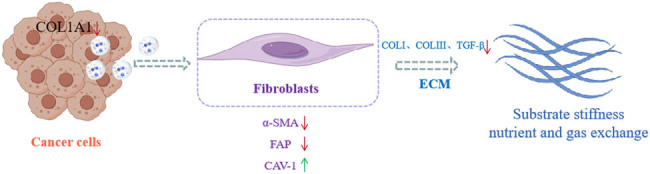

## 1 Introduction

Breast cancer is a common malignant tumor in women, which can seriously threaten their health. With the development of technologies for tumor diagnosis and treatment, the treatment efficacy and survival rate of breast cancer patients have been gradually improved [1]. However, the recurrence and drug resistance of breast cancer have greatly limited the treatment efficacy and survival of patients [2]. Therefore, it is important to reveal the pathogenesis of breast cancer.

The tumor microenvironment is a complex tissue environment that can regulate tumor growth and metastasis [3]. There are a variety of stromal cells in the tumor microenvironment. Tumor-associated fibroblasts represent an important part of the tumor microenvironment, which can secrete a variety of degrading enzymes, maintain the stability of the extracellular matrix, increase the expression of vascular endothelial growth factor, promote angiogenesis, and eventually lead to the development of tumors [4,5,6,7]. The remodeling of the tumor microenvironment matrix is a key factor affecting the occurrence and development, drug resistance, immune escape, and distant metastasis of tumors [8]. Collagen type I alpha 1 (COL1A1), as an important component of tumor microenvironment, encodes the precursor α1 chain of type I collagen (COL Ⅰ), which forms a triple helix composed of two α1 chains and one α2 chain. COL Ⅰ, which forms fibrous structures, is abundant in various connective tissues. It has been reported that there is increased expression of COL1A1 in many tumors, which can participate in matrix remodeling and promote tumor invasion and distant metastasis [9,10,11]. The expression of COL1A1 in breast cancer tissues serves as a valuable reference for assessing malignant progression and prognosis, as well as guiding targeted therapy in breast cancer patients. Therefore, there may be a regulatory relationship between the COL1A1 and tumor microenvironment matrix remodeling.

Exosomes, about 30–100 nm in size, are nanovesicles derived from membranes. They can be secreted by various cells, including mast cells, B lymphocytes, and nerve cells. In particular, tumor cells can produce and secrete more exosomes than normal cells. The exosomes are rich in nucleic acids (miRNA, lncRNA, circRNA, mRNA, etc.), proteins, and cholesterols. Exosomes play a dual role in the tumor microenvironment, as they enable cancer cells to manipulate immune cells for the promotion of tumor growth and survival [12]. In contrast, immune cells can utilize exosomes to activate immune responses in the tumor microenvironment, consequently impeding tumor cell growth [13]. Studies [14,15] have shown that cancer cells can release exosomes containing COL1A1, which subsequently travel to metastatic sites via the circulatory system. Fibroblasts at the metastatic site uptake these exosomes and undergo activation, transforming into cancer-associated fibroblasts that secrete inflammatory factors [16]. This process enhances tumor cell epithelial–mesenchymal transition, promotes stemness, and contributes to the formation of a niche that facilitates tumor metastasis [17]. However, the role of COL1A1 in tumor-associated fibroblast activation and matrix remodeling in the tumor microenvironment of breast cancer remains largely known.

In this study, the role and mechanism of COL1A1 in inducing fibroblasts to transform into tumor-associated fibroblasts and promoting matrix remodeling of breast cancer was investigated. Our findings may provide a theoretical basis for the further use of exosomes in constructing tumor-suppressing vectors.

## 2 Materials and methods

### 2.1 Cell line and cell culture

Human breast epithelial cell line MDA MB-231 (Guangzhou Huatuo Biotechnology Co., Ltd., Guangzhou, Guangdong, China) was cultured with Dulbecco’s modified eagle medium containing 10% fetal bovine serum (Shanghai Ecosai Biological Products Co., Ltd., Shanghai, China). The fibroblast HKF cells (Beijing Fenghui Biotechnology Co., Ltd., Beijing, China) were cultured with minimum essential medium containing 10% fetal bovine serum and 1% nonessential amino acid, in a 37°C, 5% CO_2_ incubator. Cell passaging was performed when 80% confluence was achieved.

### 2.2 The siRNA transfection

The MDA-MB-231 cells were transfected with siRNA-COL1A1 or siRNA negative control using lipofectamine RNAiMAX, according to the instructions. According to different treatments, the cells were divided into a blank control group (without transfection), a negative control group (transfected with siRNA negative control), and a siRNA-COL1A1 group (transfected with siRNA-COL1A1). The transfected cells were incubated in a 37°C, 5% CO_2_ incubator for 48 h.

### 2.3 Extraction and observation of exosomes

After transfection for 48 h, the culture supernatant of MDA-MB-231 cells was collected. The exosomes were extracted from the cell culture supernatant by differential centrifugation. The dead cells were removed by 3,000×*g* centrifugation at 4°C for 15 min. Then, the cell debris was removed by 6,000×*g* centrifugation at 4°C for 40 min. Then, the supernatant was collected after 10,000×*g* centrifugation at 4°C for 40 min. After 100,000×*g* centrifugation at 4°C for 60 min, the precipitate was collected, which was the exosome. The exosomes were then re-suspended and analyzed with the Nano Measurer software. The particle size and distribution of exosomes were determined.

### 2.4 Cell co-culture with exosomes

HKF cells were co-cultured with exosomes extracted from MDA MB-231 cells. Briefly, HKF cells in the HKF control group were cultured without any intervention. For the HKF + EXO, HKF + siRNA negative control-EXO, and HKF + siRNA-COL1A1-EXO groups, HKF cells were co-cultured with 50 μg/mL exosomes, which were respectively isolated from the normally cultured MDA-MB-231 cells, MDA-MB-231 cells transfected with siRNA negative control, and MDA-MB-231 cells transfected with siRNA-COL1A, for 24 h.

### 2.5 Western blot analysis

Protein expression levels in the exosomes and fibroblasts were detected with Western blot analysis. Briefly, the exosomes or fibroblasts were lysed to extract total proteins. The protein concentration was determined with the bicinchoninic acid method. The protein sample was separated with the sodium dodecyl sulfate-polyacrylamide gel electrophoresis and electronically transferred onto the membrane. After blocking, the membrane was incubated with the rabbit anti-CD63 (bs-1523R; Bioss, Beijing, China), rabbit anti-CD81 (bs-6934R; Bioss), rabbit anti-Collagen I (bs-10423R; Bioss), rabbit anti-E cadherin (bs-10009R; Bioss), rabbit anti-Caveolin-1 (Cav-1) (bs-1453R; Bioss), and anti-fibroblast activation protein (FAP) (PB0096; Boster, Wuhan, Hubei, China) primary antibodies, respectively, at 4°C overnight. After washing, the membrane was incubated with goat anti-rabbit IgG H&L (HRP) (ab205718; Abcam) or goat anti-mouse IgG H&L (HRP) (ab205719; Abcam) secondary antibodies, at room temperature for 2 h. Then, the protein bands were visualized and analyzed with the ChemiScope mini chemiluminescence instrument. β-Actin was used as an internal reference.

### 2.6 Enzyme-linked immunosorbent assay (ELISA)

The co-cultured fibroblasts were collected and lysed. Then, the contents of COL Ⅰ, COL Ⅲ (type Ⅲ collagen), and TGF-β1 in the lysate were determined with the Human Collagen Type I ELISA Kit (CSB-E08082h; Cusabio, Wuhan, Hubei, China), Human Collagen Type III ELISA Kit (CSB-E04799h; Cusabio), and the Human/Mouse/Rat TGF-β1 ELISA Kit (70-EK981-48; Cusabio). Briefly, the lysate was mixed with carbonate buffer, which was then added to the detection plate and incubated at 4°C overnight. After rinsing, 1% bovine serum albumin was added and incubated at 37°C for 60 min. Then, 0.1 mL antiserum was added for incubation at 37°C for 40 min. After washing, 3,3′,5,5′-tetramethylbenzidine color development was performed. Finally, the optical density value at 450 nm was detected, and contents of COL Ⅰ, COL Ⅲ, and TGF-β1 were determined.

### 2.7 Quantitative real-time polymerase chain reaction (PCR)

Total RNAs were extracted from the fibroblasts and reverse transcribed into cDNA. The mRNA expression levels were detected with the quantitative real-time PCR. The primers were designed using Primer-BLAST software [18] and their sequences are presented in Table 1. The 20-μL reaction system consisted of 0.4 μL primer each, 1 μL cDNA template, 10 μL SYBGreen (MasterMix-LR; Abcam), and distilled water. The PCR conditions were set as follows: 95°C for 3 min; 95°C for 15 s, 60°C for 40 s, for 40 cycles; and 72°C for 10 min. The expression levels of target genes were calculated with the 2^−△△Ct^ method.

**Table 1:** The primer sequences

Gene	Forward primer	Reverse primer
*E-cadherin*	5′-ATCATGTTTGAGACCTTCAACA-3′	5′-CATCTCTTGCTCGAAGTCCA-3′
*α-SMA*	5′-GTTTACCTACTCGTCTCTGGTAC-3′	5′-CTTATCCCAATACGTGTCGACAT-3′
*FAP*	5′-TCAGTGTGAGTGCTCTCATTGTAT-3′	5′-GCTGTGCTTGCCTTATTGGT-3′
*Cav-1*	5′-TGGGTCTGTTATTGATGAGCC-3′	5′-TGACTTCCTTCCATTCTGAAGAC-3′
*GAPDH*	5′-CGTTGATTAAGTCCCTGCCCTT-3	5′-TCAAGTTCGACCGTCTTCTCAG-3′

### 2.8 Statistical analysis

Data were expressed as mean ± standard deviation (SD). SPSS26.0 software was used for statistical analysis. The *F* test was used for comparison among multiple groups. *P* < 0.05 was considered statistically significant.


**Ethical approval:** The conducted research is not related to either human or animals use.

## 3 Results

### 3.1 Confirmation of the exosomal nature of the extracted vesicles

**Figure 1:**
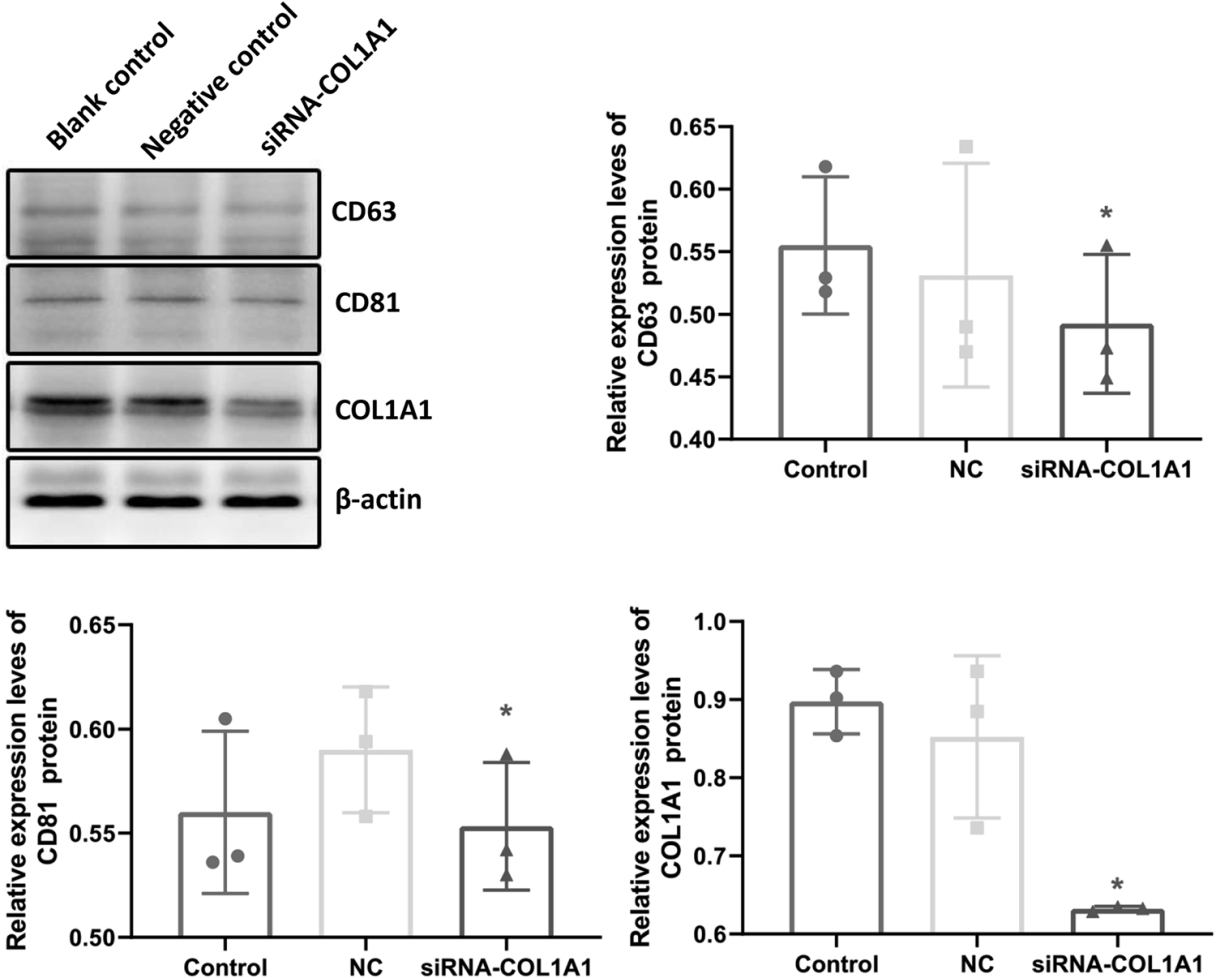
Protein expression levels in cancer cell exosomes. The protein expression levels of CD63, CD81, and COL1A1 in the cancer cell exosomes were detected by Western blot analysis. Representative and quantitative Western blot results were shown. Compared with blank control, *P < 0.05; compared with negative control, ^#^ *P* < 0.05.

CD63 and CD81 proteins are specific markers for exosomes [19]. To determine the exosomal nature of the extracted vesicles and the presence of COL1A1 in exosomes, the Western blot analysis of CD63, CD81, and COL1A1 was performed. As shown in Figure 1, there was no statistical difference in the protein expression levels of CD63 and CD81 among these groups (*P* > 0.05). Compared with the blank control group and the negative control group, the protein expression level of COL1A1 in the siRNA-COL1A1 group was significantly decreased (*P* < 0.05). This result confirmed the exosomal nature of the extracted vesicles.

### 3.2 Characterization of cancer cell exosomes

The cancer cell exosomes were further identified. As shown in Table 2, there was no statistical difference between the average particle size and total concentration among these groups (*P* > 0.05). Compared with the blank control group and the negative control group, the main peak of particle size and the SD value of particle size 30–200 nm in the siRNA-COL1A1 group were significantly increased, while the percentage of particle size 30–150 nm, the SD value of particle size 30–150 nm, and the percentage of particle size 30–200 nm were significantly decreased (*P* < 0.05). These results suggest that the siRNA-COL1A1 inhibits the secretion of exosomes by breast cancer cells.

**Table 2:** Identification of cancer cell exosomes with transmission electron microscope

	Dilution multiple	Average particle size (nm)	Main peak of particle size (nm)	Total concentration (1 × 10^8^/mL)	Percentage of particle size 30–150 nm (%)	SD value of particle size 30–150 nm	Percentage of particle size 30–200 nm (%)	SD value of particle size 30–200 nm
Blank control	50	207.90	183.80	13.70 ± 0.48	7.85	4.13	52.79	2.16
Negative control	50	221.80	178.30	15.00 ± 0.72	8.78	8.56	47.44	1.50
siRNA-COL1A1	50	213.50	247.20^△^▲	14.60 ± 0.497	7.18^△^▲	1.81	43.62^△^▲	5.21

Note: Compared with blank control, ^△^ *P* < 0.05; and compared with negative control, ▲*P* < 0.05.

### 3.3 Contents of COL Ⅰ, COL Ⅲ, and TGF-β1 in fibroblasts

**Figure 2:**
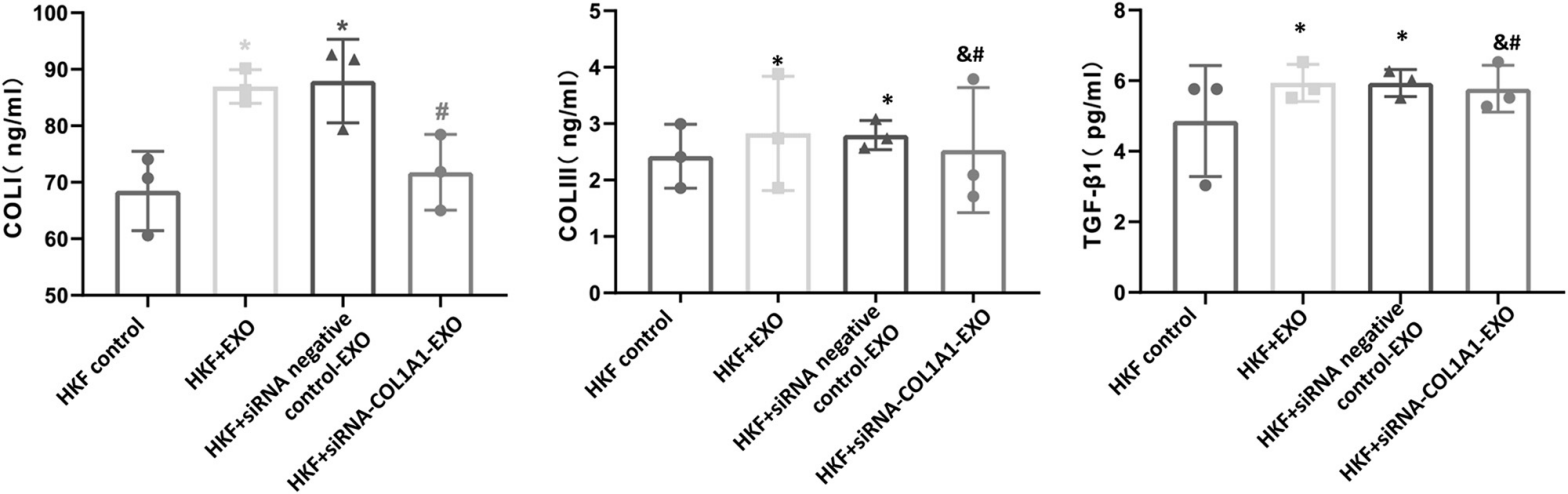
Contents of COL Ⅰ, COL Ⅲ, and TGF-β1 in fibroblasts. Levels of COL Ⅰ, COL Ⅲ, and TGF-β1 in fibroblasts were analyzed with ELISA. Compared with HKF control, *P < 0.05; compared with HKF + EXO co-culture, ^#^ *P* < 0.05; and compared with HKF + siRNA negative control-EXO co-culture, ^&^ *P* < 0.05.

To evaluate the fibrosis in the tumor microenvironment, the contents of COL Ⅰ, COL Ⅲ, and TGF-β1 in fibroblasts were analyzed with ELISA. As shown in Figure 2, compared with the HKF control group, the contents of COL Ⅰ, COL Ⅲ, and TGF-β1 in the HKF + EXO co-culture group and HKF + siRNA negative control-EXO co-culture group were significantly increased (*P* < 0.05). Compared with the HKF + EXO co-culture group and HKF + siRNA negative control-EXO co-culture group, the contents of COL Ⅰ, COL Ⅲ, and TGF-β1 in the HKF + siRNA-COL1A1-EXO co-culture group were significantly decreased (*P* < 0.05). These results suggest that the COL1A1 silencing inhibits the release of COL Ⅰ, COL Ⅲ, and TGF-β1 from fibroblasts.

### 3.4 Gene expression levels in fibroblasts

**Figure 3:**
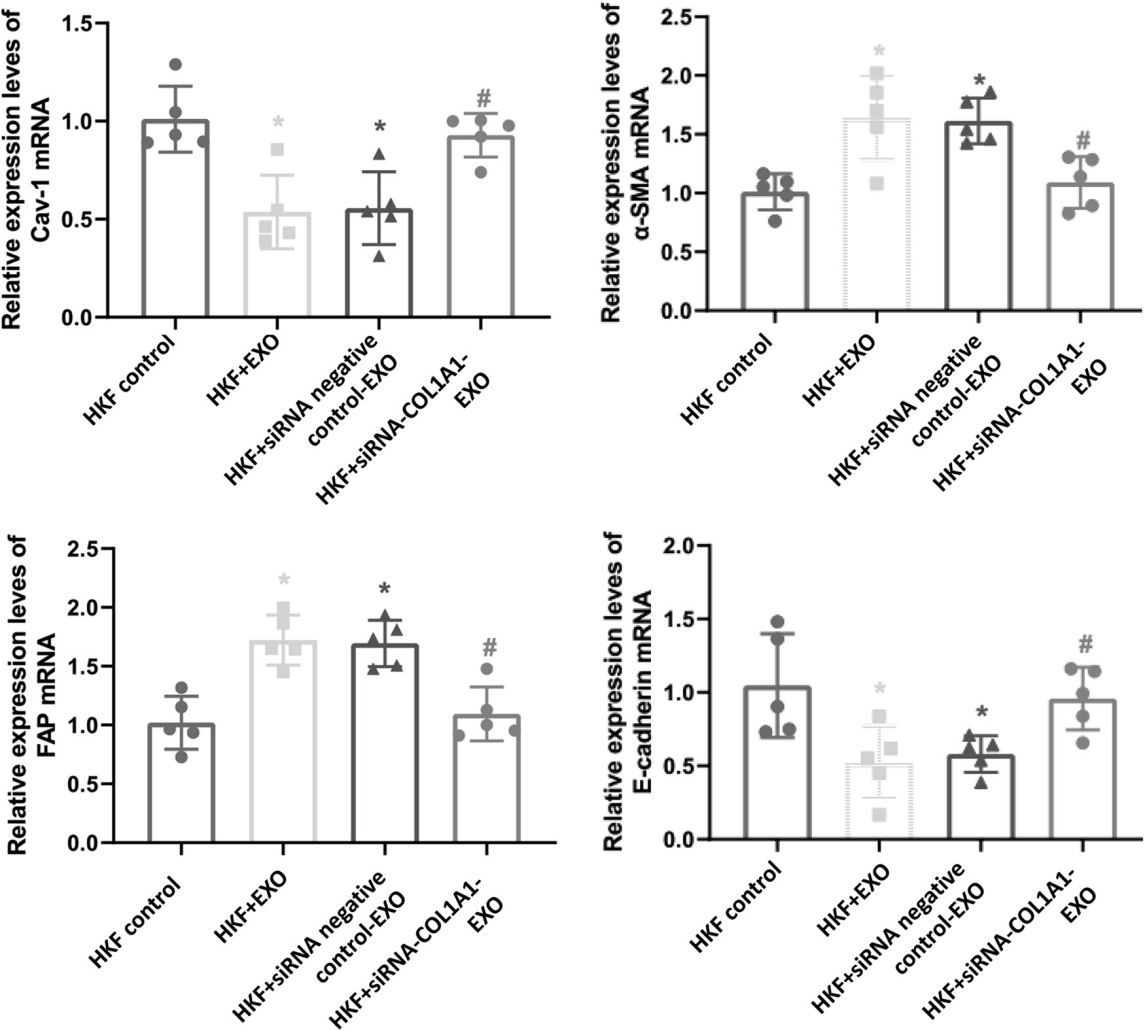
Gene expression levels in fibroblasts. The mRNA expression levels of *E-cadherin*, *α-SMA*, *FAP*, and *Cav-1* in the fibroblasts were detected by quantitative real-time PCR. Compared with HKF control, *P < 0.05; compared with HKF + EXO co-culture, ^#^ *P* < 0.05; and compared with HKF + siRNA negative control-EXO co-culture, ^&^ *P* < 0.05.

The quantitative real-time PCR was performed to assess the expression of fibrosis-related genes (including *E-cadherin*, *α-SMA*, *FAP*, and *Cav-1*) in the fibroblasts of the tumor microenvironment. As shown in Figure 3, compared with the HKF control group, the mRNA expression levels of *E-cadherin* and *Cav-1* in the HKF + EXO co-culture group and HKF + siRNA negative control-EXO co-culture group were significantly decreased, while the mRNA expression levels of *α-SMA* and *FAP* were significantly increased (*P* < 0.05). Compared with the HKF + EXO co-culture group and HKF + siRNA negative control-EXO co-culture group, the mRNA expression levels of *E-cadherin* and *Cav-1* in the HKF + siRNA-COL1A1-EXO co-culture group were increased, whereas the expression levels of *α-SMA* and *FAP* were significantly decreased (*P* < 0.05).

### 3.5 Protein expression levels in fibroblasts

**Figure 4:**
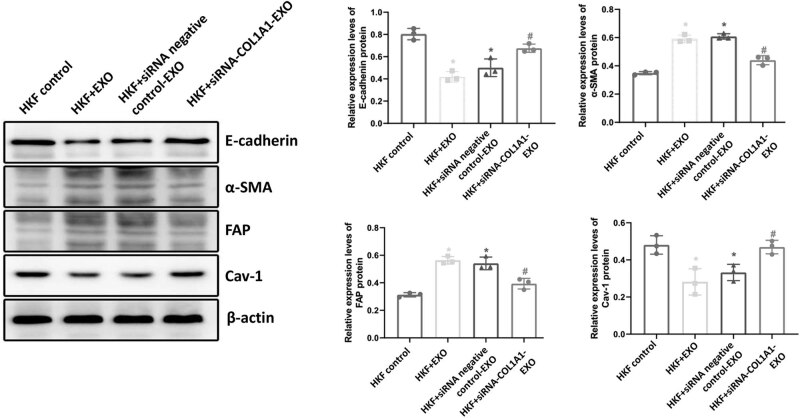
Protein expression levels in fibroblasts. The protein expression levels of E-cadherin, α-SMA, FAP, and Cav-1 in the fibroblasts were detected by Western blot analysis. Representative and quantitative Western blot results were shown. Compared with HKF control, *P < 0.05; compared with HKF + EXO co-culture, ^#^ *P* < 0.05; and compared with HKF + siRNA negative control-EXO co-culture, ^&^ *P* < 0.05.

After COL1A1 knockout, Western blot was employed to examine the expression of fibrosis-related proteins (E-cadherin, α-SMA, FAP, and Cav-1) in the tumor microenvironment, aiming to determine any alterations in matrix fibrosis levels. As shown in Figure 4, compared with the HKF control group, the protein expression levels of E-cadherin and Cav-1 in the HKF + EXO co-culture group, HKF + siRNA negative control-EXO co-culture group, and HKF + siRNA-COL1A1-EXO co-culture group were significantly decreased, while the protein expression levels of α-SMA and FAP were significantly increased (*P* < 0.05). Compared with the HKF + EXO co-culture group and HKF + siRNA negative control-EXO co-culture group, the protein expression levels of E-cadherin and Cav-1 in the HKF + siRNA-COL1A1-EXO co-culture group were significantly increased, while the protein expression levels of α-SMA and FAP were significantly decreased (*P* < 0.05). These results revealed that after COL1A1 silencing, the expression levels of ɑ-SMA and FAP were decreased, while the expression levels of E-cadherin and Cav-1 were increased, indicating that COL1A1 silencing may inhibit fibroblast activation and tumor metastasis.

## 4 Discussion

The tumor microenvironment plays an important role in regulating the occurrence and development of tumors and participates in tumor growth, metastasis, and immune escape [20]. The tumor microenvironment is composed of multiple components, including immune cells, endothelial cells, and fibroblasts. The most important stromal cells are tumor-associated fibroblasts, which can directly contact tumor cells to accelerate the occurrence and development of tumors [21].

Tumor exosomes are extracellular capsules with lipid bilayer membranes. The main marker proteins of tumor exosomes are CD63 and CD81, which are widely found in the peripheral blood and body fluid [22,23]. They can regulate the occurrence and development of tumors in various ways [24,25]. Breast cancer exosomes can activate tumor-associated fibroblasts to accelerate the invasion and metastasis of tumors [26,27,28]. In this study, our results showed that the down-regulation of COL1A1 could reduce CD63 and CD81 levels in exosomes and inhibit the production of exosomes. Previous findings have shown that [29,30,31] exosomes can transform normal breast epithelial cells, induce human breast epithelial cells to produce reactive oxygen, damage DNA, induce normal epithelial cells to undergo malignant transformation, and promote the growth and proliferation of breast cancer cells. Tumor-associated fibroblasts in the tumor microenvironment could secrete a large number of exosomes, which activate the proliferation signal pathway of breast cancer cells, thereby promoting tumor proliferation and inducing the occurrence of breast cancer [32,33]. In line with these findings, our results herein showed that the COL1A1 down-regulation may inhibit the activation of tumor-related fibroblasts and reduce the production of exosomes.

Collagen is the main component of the extracellular matrix. The change in collagen can lead to many kinds of fibrotic diseases [34,35]. COL I can transmit exogenous stimulating signals to cells through integrin, glycoprotein, and other transmembrane receptors [36,37]. Moreover, COL I can transport growth factors, playing important roles in wound healing, tissue and organ development, and tissue repair [38,39]. TGF-β1 can regulate the production and deposition of the extracellular matrix and degrade the extracellular matrix by up-regulating the activation factor of the fibrinolytic enzyme, to accelerate the migration of tumor cells [40,41]. In this study, our results indicated high expression levels of COL Ⅰ, COL Ⅲ, and TGF-β1 in breast cancer cells. Moreover, down-regulated expression of COL1A1 reduced COL Ⅰ, COL Ⅲ, and TGF-β1 in fibroblasts. Previous studies have shown that [42,43] TGF-β1 is important in the development of fibrotic diseases, which can promote the biosynthesis of COL Ⅰ collagen, and thus plays an important role in the process of tissue repair. Moreover, COL Ⅰ can bind with decorin to block the effect of TGF-β1 in the tissues [44,45]. In line with these findings, our results herein showed that down-regulation of COL1A1 expression may inhibit the production of COL Ⅰ, COL Ⅲ, and TGF-β1 by fibroblasts.

The abnormal expression of E-cadherin is an important sign of epithelial–mesenchymal transformation. The reduced expression of E-cadherin can cause epithelial–mesenchymal transformation and accelerate the metastasis and invasion of tumor cells [46,47]. The α-SMA is a common molecular marker for the activation of interstitial fibroblasts [48,49,50]. With the development of tumors, fibroblasts in the stroma are constantly activated to improve the local microenvironment of breast cancer [51,52]. Cav-1 plays an important role in signal transduction, lipid transport, and cholesterol homeostasis. It can also promote interstitial remodeling of fibroblasts and affect the microenvironment of tumor cells [53,54]. In this study, our results showed that down-regulation of COL1A1 increased the expression of E-cadherin and Cav-1 in tumor cells and reduced the expression of α-SMA and FAP, thus improving the tumor microenvironment. Previous findings have shown that [55] down-regulation of COL1A1 can increase the expression of E-cadherin, enhance the adhesion between cells, maintain the epithelial cell matrix, accelerate the apoptosis of tumor cells, and inhibit the growth of tumors. Consistently, our results herein showed that down-regulation of COL1A1 improved the tumor microenvironment and inhibited the activation of fibroblasts.

This study has some limitations. First, the study results were solely based on cellular experiments. No validation with animal experiments was conducted. Second, the specific cellular pathways underlying the effects of COL1A1 on breast cancer were not further investigated in this article. Further studies are warranted.

In conclusion, the gene COL1A1 may potentially function as an oncogene in breast cancer. Its down-regulation has the potential to inhibit the activation of tumor-associated fibroblasts and the remodeling of the extracellular matrix in the tumor microenvironment, thereby inhibiting tumor development. This inhibition may be achieved by suppressing the secretion of exosomes, reducing the release of COLⅠ, and upregulating the expression of CAV-1. Moving forward, we aim to delve deeper into the mechanisms that underlie these effects. Our findings may contribute valuable evidence to the development of novel anti-tumor therapies for breast cancer.
